# Clinical manifestations and outcomes of severe malaria in adult patients admitted to a tertiary hospital in the Gambia

**DOI:** 10.1186/s12936-022-04294-4

**Published:** 2022-09-21

**Authors:** Sheikh Omar Bittaye, Abubacarr Jagne, Lamin ES Jaiteh, Behzad Nadjm, Alfred Amambua-Ngwa, Abdul Karim Sesay, Yankuba Singhateh, Emmanuel Effa, Ousman Nyan, Ramou Njie

**Affiliations:** 1 Department of Internal Medicine, Edward Francis Small teaching hospital, Banjul, The Gambia; 2 School of Medicine and Allied Health Sciences, University of The Gambia, Banjul, The Gambia; 3 Medical Research Council, London School of Hygiene and Tropical Medicine, Fajara, The Gambia; 4 Epidemiology and disease control unit, Ministry of Health, Banjul, The Gambia

**Keywords:** Clinical features, Outcome, Severe malaria, Adult, Gambia

## Abstract

**Background:**

Malaria is a major public health concern in The Gambia. There is limited data on the clinical manifestation and outcome of severe malaria in adult patients in The Gambia. The study therefore assessed the clinical manifestations and outcome of severe malaria in adult patients admitted at the Edward Francis Small Teaching Hospital.

**Methods:**

The study retrospectively reviewed the records of all malaria patients admitted from 18th October 2020 to 2nd February 2022. Demographic data, clinical features, investigations, treatment, and outcomes were recorded.

**Results:**

A total of 131 confirmed malaria patients were recruited into the study. The median age was 21 yrs, range (15–90) and most of them were within the youth age group (15–24yrs) 85 (64.9%). The majority of the patients were also male 88 (67.2%) with a male to female ratio of 2:1. The most common symptom at presentation was fever 119 (90.8%) and the most common sign was pallor 48 (36.6%). Seventy-six patients (58.1%) and 55 (41.9%) patients met the criteria for severe malaria and uncomplicated malaria diagnosis, respectively. The most common clinical feature amongst patients with severe malaria were impaired consciousness 34 (44.7%), severe anaemia 26 (34.2%) and acute kidney injury 20 (26.3%). Patients with severe malaria were younger with mean age of 22.9 vs. 29 yrs (p = 0.004), more likely to be referred from a lower-level health facility 62 (81.6%) vs. 34 (61.8%) (p = 0.012), to have a longer duration of admission (p = 0.024) and to die 13 (17.1%) vs. 0 (0%) (p = 0.001) as compared to patients with uncomplicated malaria. The total mortality was 13 (9.9%) and all the patients who died had severe malaria. Mortality was higher in patients with impaired consciousness 9 (26.5%) and there was a significant relationship between death and impaired consciousness 9 (69.3%) vs. 25 (21.4%) p = 0.001.

**Conclusion:**

Severe malaria still affects young adults in an endemic area with significant mortality. This suggests the need for targeted malaria prevention, surveillance, case management and control strategies in this population group in The Gambia to help reduce morbidity and mortality of malaria.

## Background

In malaria elimination settings, spatial and temporal heterogeneity in incidence, and hotspots of transmission become increasingly prevalent as disease burden declines [1]. In sub-Saharan Africa, which bears the highest burden of malaria, *Plasmodium falciparum* malaria remains a major cause of disease and death, particularly in young children because a substantial proportion of older children and adults have acquired protective immunity. As intensity of transmission reduces, the age immunity patterns change and older children may become increasingly susceptible to severe malaria, even though the overall malaria burden may continue to decline [2, 3].

Malaria in The Gambia is seasonal with peak incidence of clinical cases and mortality between September and November [4]. It also affects the entire population and is a leading cause of morbidity and mortality, especially among children under 5 years [5]. However, there has been a steady decline of malaria as indicated by a downward trend in slide positivity rates at health facilities, and unprecedented low incidence and seroprevalence in community surveys [6]. The annual malaria incidence has also declined by 43% across all seven regions over the past four years from 149 to 1000 population in 2011 to 74 in 2016 [5]. When the area/location of a patient is coupled with clinical data, it may be possible to identify populations or areas that carry the heaviest burden of malaria or are the most important potential contributors to malaria transmission [7]. Consequently, the impact of malaria control efforts can be maximized by implementing tailored control measures to carefully selected areas and population [8].These interventions could also be perturbed by other public health events, such as covid-19, which could affect the incidence and disease presentation.

Clinical manifestations of malaria vary between children and adults. Signs and symptoms can range from mild febrile syndrome in uncomplicated malaria to life-threatening conditions with severe anaemia, acute respiratory distress syndrome, hypoglycaemia, shock, metabolic acidosis, acute kidney injury, and/or cerebral malaria in patients with severe malaria [9, 10]. Severe malaria is common in children in areas with high transmission [11, 12], whereas in areas with low transmission, all age groups are equally affected [13–15]. Cerebral malaria, acute kidney injury, severe jaundice and pulmonary oedema are the main manifestations in young adults but yet still the determinants of severe malaria and its pathophysiology are not completely understood [16]. Therefore, this epidemiological study on severe malaria may provide better understanding of the commonest clinical features and its related outcomes in Gambian adults.

Although severe malaria cases are commonly observed in children in The Gambia [12], there are limited published clinical studies on severe malaria in Gambian adult population. The aim of the present study is to describe the clinical manifestations, outcomes, and determinants of mortality of severe malaria in adult patients admitted at the internal medicine department of Edward Francis Small teaching hospital (EFSTH). The study therefore assessed the clinical features and outcome of severe malaria in Gambian adult patients admitted in the Internal Medicine Department of EFSTH. The morbidity and mortality data from this study will be of considerable value for the early identification of individuals who are at risk of becoming critically ill or dying and who are most likely to benefit from early preventive measures and possible treatment.

## Methods

This was a retrospective descriptive hospital-based study conducted at the only tertiary hospital in The Gambia, EFSTH, Banjul, from the 18th October 2020 to 2nd February 2022. EFSTH has a total bed capacity of 500. It is the major referral centre in the country and receives patients from the whole country. The hospital offers specialists clinical services in most areas and is a centre for research and training for medical students, house and medical officers and residents. The hospital also has an accident and emergency unit and an intensive care unit.

This study assessed the clinical manifestations and outcome of severe malaria in adult patients admitted at the EFSTH, Banjul, The Gambia. Severe malaria was defined according to the World Health Organization (WHO) criteria, namely, the presence of *Plasmodium falciparum* in the thick blood film (BF) or positive rapid diagnostic test (RDT) or both, in association with one or more major criteria of severity [2]. These criteria included impaired consciousness (Glasgow coma score < 11), convulsions (more than two per 24 h), shock (systolic blood pressure < 80 mmHg with cold extremities), severe anaemia (haemoglobin < 7 gm/dl), jaundice (plasma or serum bilirubin > 50 µmol/L (3 mg/dL) with a parasite count > 100 000/ µL), renal impairment (plasma or serum creatinine > 265 µmol/L (3 mg/dL) or blood urea > 20 mmol/L), acidosis (base deficit of > 8 mEq/L or a plasma bicarbonate level of < 15 mmol/L or venous plasma lactate ≥ 5 mmol/L), hypoglycaemia (blood glucose < 2.2mmol/dl) and hyperparasitaemia (*P. falciparum* parasitaemia > 10%), significant bleeding (recurrent or prolonged bleeding from the nose, gums or venepuncture sites; haematemesis or melaena) as described by WHO guidelines, and the rest were considered as uncomplicated cases. Convulsions were noted at presentation but there was no seizure chart for continuous monitoring. Hyperparasitaemia was not assessed as it was qualitatively determined in < 20% of patients. Jaundice and acidosis were also not assessed due to limited laboratory resources. Youth was defined as the age group between 15 and 24 years and adult above 24 years [17].

Laboratory diagnosis of malaria was by a rapid diagnostic test (RDT; paraHIT^®^ -f Ver1.0, Arkray, Gujarat, India) from capillary blood and/ or thick blood film (BF) prepared from capillary blood, stained with Giemsa, examined under 100 oil immersion microscopy. Haemoglobin concentrations were estimated using a HemoCue haemoglobinometer (HemoCue 301, Angelhom, Sweden). Blood glucose was measured at presentation in all patients using the bed side device Accu-Chek®active (Roche Diagnostics, Mannheim Germany).

All RDT and/or BF confirmed malaria patients admitted into the internal medicine department of EFSTH were recruited into the study. The study used a structured questionnaire to extract secondary data from the patient’s records, which included referral facility, demographic data, symptoms, signs, laboratory investigations, treatment and clinical outcomes. All patients of at least 15 years of age with laboratory confirmed diagnosis of malaria by RDT and/or BF and admission to the Internal Medicine department of EFSTH were included in the study. Malaria infected patients with underlying severe chronic cardiac, renal, hepatic diseases or human immunodeficiency virus /acquired immunodeficiency syndrome or cerebrovascular disease, which may interfere with the evolution of malaria, and pregnant women, were excluded from analysis. The study excluded 9 patients (1 patient with incomplete data, 4 patients with cerebrovascular accident, 1 congestive heart failure, 1 chronic kidney disease patient and 2 with chronic liver disease).

Patients with severe malaria, or those with non-severe disease unable to tolerate oral medication, were treated with intravenous infusion of artesunate 2.4 mg/kg at 0 h, 12 and 24 h and thereafter, administered once daily until the patient is able to tolerate oral anti-malarial (artemether-lumefantrine) therapy as recommended by the WHO [2]. Due to the increased number of malaria cases in 2021–2022 there was a stock out of artesunate and thus intravenous quinine was used in small number of cases (Table 1). When the patients were able to swallow, parenteral treatment was discontinued and a three-day oral artemether-lumefantrine treatment given. Resuscitation and supportive management were given according to WHO guidelines [2] including correction of hypoglycaemia with 50% glucose, termination of convulsions with intravenous diazepam, blood transfusion for those with severe anaemia (haemoglobin < 7 g/dl) and haemodialysis for patients with acute kidney injury.

**Table 1:** Baseline characteristics of patients with malaria in EFSTH

Variable	All n = 131 (%)
Age: Median (yrs) (range)	21 (15–90)
Age groups (yrs)	
15–24 (Youth)	85 (65)
> 24 (Adult)	46 (35)
Sex (M:F)	88 (67.2):43 (32.8)
Type of referral Self referral Health facility • Kanifing General hospital • Bundung maternal and child hospital • Brikama district hospital • Fajikunda health centre • Essau district hospital • Others	35 ( 26.7) 96 (73.3) 30 (31.3) 23 (23.9) 15 (15.6) 14 (14.5) 4 (4.2) 8 (8.3)
Prior antimalarial treatment for referred patients	n = 96
No medication	42 (43.8)
Intravenous artesunate	53 (55.2)
Oral Artemether-lumefantrine	1 (1)
Symptoms at presentation	
Fever	119 (90.8)
Headache	106 (80.9)
Vomiting	96 (73.3)
Malaise	39 (29.8)
Abdominal pain	50 (38.2)
Convulsion	21 (16)
Signs at presentation	
Pallor	48 (36.6)
Jaundice	38 (29)
Median Glasgow coma score at presentation	14 (3–15)
Treatment and outcome	
Treatment	
Antimalaria at presentation • Intravenous artesunate • Intravenous Quinine • Oral Artemether-lumefantrine	123 (93.9) 2 (1.5) 6 (4.6)
Intravenous fluid therapy	117 (89.3)
Anti seizure medication	14 (10.7)
Antibiotics	45 (34.3)
Blood transfusion	17 (12.9)
Haemodialysis	9 (6.9)
• Alive	8
• Dead	1
Outcome	
Dead	13 (9.9)
Alive	118 (90.1)
Duration of admission: Median (hours) (range)	72 (4–552)

Data was entered into a Microsoft Excel database (Microsoft Corp, Redmond, WA, USA) which was imported to and analysed using STATA/SE 14.2 (Statacorp, TX, USA). Simple proportion was calculated for discrete variables. Chi-squared test and Fisher’s exact test were used for discrete variables and Mann-Whitney U-test and Students t-test were used for continuous variables with skew and normal distribution respectively. Statistical significance was defined as p < 0.05.

## Results

### Clinical characteristics of the study population

**Fig. 1:**
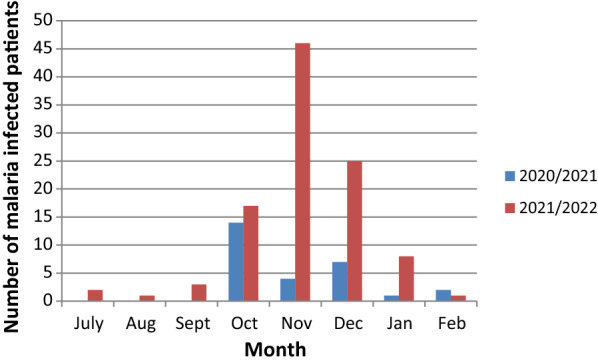
The number of patients with malaria admitted in EFSTH, in 2020–2022 in different months

**Fig. 2:**
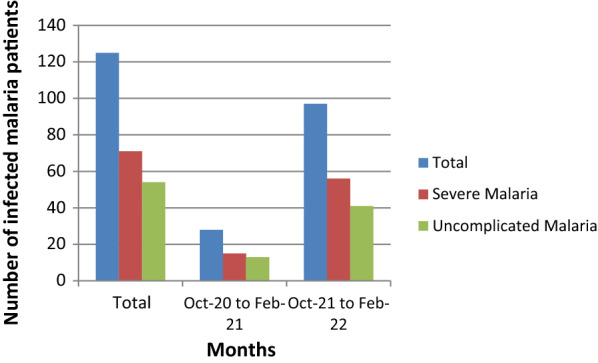
Impact of COVID 19 restrictions on malaria admissions in EFSTH during the pandemic

**Fig. 3:**
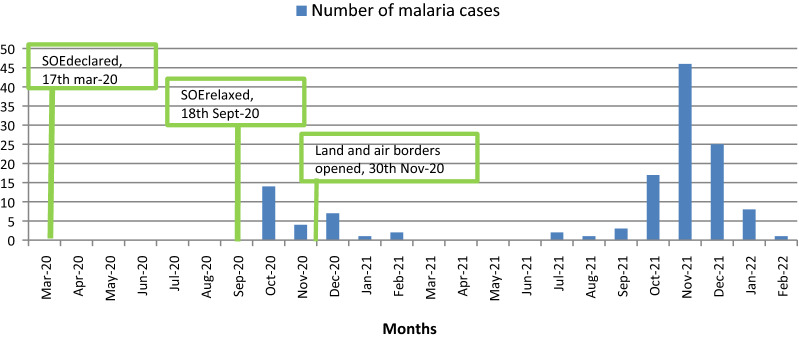
The number of patients with uncomplicated or severe malaria admitted in EFSTH, between Oct-20 to Feb-21 and Oct-21 to Feb-22

A total of 171 malaria cases (131 adults and 40 children) were admitted during the study period. The study recruited 131 adult patients with malaria. Of these, 107 (81.7%) were diagnosed by positive RDT, 4 (3%) by the presence of malaria parasites on thick blood film and 20 (15.3%) by both RDT and thick blood film. The number of malaria cases admitted in the internal medicine department in 2021 was 97 cases and admission peaked in November (Figs. 1 and 2). The number of admitted malaria cases increased from 28 patients in October 2020 to February 2021 to 97 patients in October 2021 to February 2022. This also reflected an increase in the number of both uncomplicated and severe malaria cases (Fig. 3).

The median age was 21 yrs, range (15–90). Most of these patients 85 (64.9%) were within the youth age group (15–24yrs) with only 5 (3.8%) patients above 60 years. The majority of the patients were also male 88 (67.2%) with a male to female ratio of 2:1. Ninety-six (73.3%) of the patients were referred from a lower-level health facility and most of them were started on intravenous artesunate 53 (55.2%) before referral. The most common symptom at presentation was fever 119 (90.8%) and the most common sign was pallor 48 (36.6%), (Table 1). The median duration of admission was 72 h, range (4-552).

## Clinical features of severe malaria patients

Seventy-six (58.1%) and 55 (41.9%) patients met the criteria for severe malaria and uncomplicated malaria diagnosis, respectively. Fifty-nine (77.6%) of the patients with severe malaria were in the 15–24 year age group. The most common clinical features at presentation amongst patients with severe malaria were impaired consciousness 34 (44.7%), severe anaemia 26 (34.2%) and acute kidney injury 20 (26.3%) (Table 2). None of the patients had hypoglycaemia, 2 (2.6%) patients developed shock and 3 (3.9%) patients had significant bleeding.

**Table 2:** Clinical features of severe malaria patients in EFSTH

Clinical features	No of patients who meet the criteria, n = 76 (%)	Mortality If criteria satisfied (No. of deaths/total no. of patients who meet the criteria)
Impaired consciousness (GCS < 11)	34 (44.7)	9 (26.5)
Severe anaemia (< 7 g/dl)	26 (34.2)	3 (11.5)
*Acute kidney injury (Cr > 265umol/l)	20 (26.3)	2 (10)

*Please note that due to the limited laboratory resources, renal function tests were requested when patients were referred with suspected diagnosis of acute kidney injury or present with decrease urine output. Out of the 131 patients recruited 49 patients were suspected to have AKI and had creatinine requested

Patients with severe malaria were younger 22.9 vs. 29 yrs (p = 0.004), more likely to be referred from a lower-level health facility 62 (81.6%) vs. 34 (61.8%) (p = 0.012), to have a longer duration of admission (p = 0.025) and to die 13 (17.1%) vs. 0 (0%) (p = 0.001) compared to patients with uncomplicated malaria (Table 3).

**Table 3:** clinical differences between patients with severe and uncomplicated malaria in EFSTH

Variable	Severe malaria n = 76 (%)	Uncomplicated malaria n = 55 (%)	P value
Age Mean (yrs)	22.9	29	0.004
Age groups (yrs)			
15–24	59 (77.6)	26 (47.3)	< 0.001
> 24	17 (22.4)	29 (52.7)	
Sex (M:F)	53 (69.7):23 (30.3)	35 (63.6):20 (36.4)	0.463
Type of referral			
Self referral	14 (18.4)	21 (38.2)	0.012
Health facility	62 (81.6)	34 (61.8)	
Outcome			
Dead	13 (17.1)	0 (0)	0.001
Alive	63 (82.9)	55 (100)	
Duration of admission: Median (hours) (range)	96 (4-552)	72 (4-360)	0.025

Patients in the youth age group were more likely to be male 64(75.3%) vs. 24 (52.3%) p = 0.007, to have severe malaria 59 (69.4%) vs. 17 (36.9%) p = < 0.001 and more likely to have a lower Glasgow coma score at presentation (p = 0.001) as compared to the adult age group (Table 4).

**Table 4:** clinical differences between youth and adult patients admitted with malaria in EFSTH

Variable	Youth n = 85 (%)	Adult n = 46 (%)	P value
Sex (M:F)	64 (75.3):21 (24.7)	24 (52.3):22 (47.8)	0.007
Symptoms at presentation			
Fever	79 (92.9)	40 (86.9)	0.257
Headache	70 (82.3)	36 (76.3)	0.569
Vomiting	61 (71.8)	35 (76.1)	0.594
Malaise	25 (29.4)	14 (30.4)	0.903
Abdominal pain	31 (36.5)	19 (41.3)	0.587
Convulsion	17 (20)	4 (8.7)	0.092
Signs			
Pallor	30 (35.3)	18 (39.1)	0.664
Jaundice	28 (32.7)	10 (21.7)	0.177
Glasgow coma score at presentation	13 (3–15)	15 (8–15)	< 0.001
Severe malaria features			
Impaired consciousness (GCS < 11)	30 (35.3)	4 (8.9)	0.001
Severe anaemia (≤ 7 g/dl)	20 (24.7)	6 (13.0)	0.146
Acute Kidney injury (Cr > 265umol/l)	14 (16.5)	6 (13.0)	0.603
Severe malaria	59 (69.4)	17 (36.9)	< 0.001
Outcome	10 (11.8)	3 (9.9)	0.338
Duration of admission: median (hours)(range)	72 (4-336)	72 (4-552)	0.561

## Treatment and outcome

Treatment modalities of all patients are presented in Table 1. One hundred and twenty-three (93.9%) of the patients received intravenous artesunate (2.4 mg/kg) and 2 (1.5%) received intravenous quinine treatment at presentation. Forty-five patients (34.3%) received antibiotics, 17 (12.9%) had blood transfusion and 9 (6.9%) had haemodialysis. Of those who had haemodialysis, 8 recovered kidney functions fully with an average number of 7 sessions and one patient died after 2 sessions. Patients who received blood transfusion were more likely to have longer duration of admission compared to those who did not [120 h (24–480) vs. 72 h (4–552) p < 0.001]. Patients who had haemodialysis were also more likely to have longer duration of admission compared to those who did not [288 h (144–552) vs. 72 h (4-360), p < 0.001]. One hundred and seventeen patients (89.3%) were put on intravenous fluid therapy and 14 (10.7%) patients were also on anti-seizure medications.

The total mortality was 13 (9.9%) (Table 1) and all the patients who died had severe malaria (Table 2). Mortality was significantly higher in October 2020 to February 2021 compared to October 2021 to February 2022 6 (21.4%) vs. 6 (6.2%) p = 0.016. Mortality was higher in patients with impaired consciousness 9/34(26.5%) as compared to 3/26(11.5%) patients with severe anaemia and 2/20 (10%) patients with acute kidney injury (Table 2).

There was a significant relationship of death with impaired consciousness 9 (69.3%) vs. 25 (21.4%) p = 0.001. Patients who died also had a shorter duration of admission 24 h (6–312) vs. 72 h (4–552) p = 0.017 as compared to those who were discharged. Majority 8(61.5%) died within the first 24 h of admission. More men (n = 9) died than women (n = 4) but there was no significant relationship (p = 0.868). Ten of the patients who died were between the 15–24 year age group and only 1 patient above 60 years of age died. Age was not associated with higher mortality (p = 0.431).

## Discussion

This study describes the clinical characteristics and outcome of severe malaria in adult patients in The Gambia. Malaria transmission is seasonal. In 2021, admission of adult malaria cases in EFSTH peaked in November. This aligns with the known fact that the incidence of clinical cases and mortality of malaria in The Gambia peaks between September and November, and rapidly declines thereafter [4]. This finding should alert the health authorities to prepare and concentrate medical interventions during this period of the year. The study also showed a relatively higher number of confirmed malaria cases among adults admitted in October 2021 to February 2022 as compared to October 2020 to February 2021. Mortality was also significantly higher in October 2020 to February 2021 compared to October 2021 to February 2022. This can be explained by the reduced access to health care services due to the coronavirus 19 (COVID 19) pandemic (such as the declaration of state of emergency (SOE) in the early phases of the pandemic) (Fig. 2) and the high level of fear and worry related to COVID 19 in Gambian adults [18] resulting in late presentation of patients. The findings highlight the impact of the COVID 19 pandemic in the incidence of malaria and its related mortalities. Thus, better education, sensitization and de-stigmatization of both diseases is essential, including emphasis on early care-seeking behaviour, which also needs more community participation [19].

The median age was 21 yrs and most of the cases were within the youth age group. Most of the admitted malaria cases in the hospital during the study period were also adults. Many factors may have contributed to the increasing number of cases in the adults. For many endemic infectious diseases, decreases in transmission has resulted in an age shift in incidence of infection towards older children [20]. As already indicated there is a downward trend in slide positivity of malaria at health facilities, and unprecedented low incidence and seroprevalence in community surveys [1, 6, 21, 22] in The Gambia. Therefore, this supports the view that adults seem to be at increased risk of clinical episodes of malaria presumably due to the waning of anti-malarial immunity that follows decreased exposure to parasites [2, 20, 23]. Other studies have also indicated a shift in the burden of malaria from younger to older individuals following implementation of successful control interventions [20]. Sixty-two per cent of children under age 5 and 69% of pregnant women age 15–49 slept under a long-lasting insecticidal mosquito nets (LLIN) the night before the national malaria indicator survey in The Gambia [5]. LLINs intervention has also been stable over the years and Gambia has recorded successful LLINs coverage as high as 90% [4]. The increased use of LLINs in the under 5 and pregnant women in The Gambia may have resulted in decreased cases of malaria in these populations. There is, therefore, need for targeted interventions for malaria prevention, surveillance, case management and control strategies for the youth age group in The Gambia.

Majority of the patients were male with a male to female ratio of 2:1. A shift in the age distribution of malaria cases observed after control interventions seem to disproportionately affect males than females, suggesting a role of gender-based occupational or behavioural differences. In adults, behavioural factors including travel, leisure and social activities, and occupational activities such as agriculture or night-time work may have increased the risk of exposure outside the household as compared to children [20]. This finding suggests that non interventional factors in the adult, male malaria patients may have contributed to the problem.

Most patients had severe malaria manifesting as multi-organ dysfunction. Impaired consciousness, severe anaemia and acute kidney injury were common in this study. Apart from impaired consciousness the findings in this study are similar to a study done in Tanzania [10]. In Malawi, prostration and hyperparasitaemia were the most common clinical features [24] and in Sudan, hypotension, cerebral malaria and convulsion [16] were common. Cerebral malaria, acute respiratory distress syndrome and acute renal failure are frequently reported in imported malaria in non-endemic industrialized countries [14]. This suggests a variation in the commonest clinical features in different countries.

In this study, patients with severe malaria were more likely to be younger (within the youth age group), referred from a lower-level health facility and die compared to patients with uncomplicated malaria. In Thailand, patients with severe malaria were also more likely to be referred from a lower- level health facility but there was no difference in age and no in hospital mortality [25]. Patients with severe malaria were also more likely to have a longer duration of admission compared to patients with uncomplicated malaria which is also similar to the study done in Thailand [25]. Further analysis also confirms the fact that patients receiving blood transfusion or on haemodialysis were more likely to be admitted longer compared to those not receiving blood transfusion or haemodialysis.

The total mortality was 9.9% and all the patients who died had severe malaria. This is similar to imported adult malaria patients in non-endemic industrialized countries [26] but is lower when compared with the study done in Tanzania [27]. In another study that assessed severe malaria and death among children and adults, there was no evidence of an emerging significant burden of severe malaria or malaria mortality among adults [28]. Mortality was higher in patients with impaired consciousness and there was a significant relationship of death with impaired consciousness. These findings were similar to other studies [26, 27, 29]. This study suggests the prognostic importance of impaired consciousness in adult patients with malaria.

This study is limited by the fact that it is a single-centre study and the results may not necessarily be generalisable to other settings. Secondly, due to the retrospective nature and the limited access to laboratory investigations, laboratory features, such as hyperparasitaemia, jaundice and acidosis could not be assessed. The study however provides a baseline assessment of the spectrum of clinical features of malaria of varying severity in adults in The Gambia, which can be compared with other studies in the future.

## Conclusion

This study affirms that adult malaria is a significant cause of morbidity and mortality in The Gambia. Males within the youth age group are disproportionately affected, with increased risk of a fatal outcome and the strongest predictor of death is impaired consciousness. This, therefore, confirms the need for the implementation of targeted interventions to control malaria in this population group and early identification of patients at risk of dying to help reduce morbidity and mortality of malaria in The Gambia.

## Data Availability

The dataset for this publication is available on reasonable request from the corresponding author.

## Abbreviations

EFSTH: Edward Francis small teaching Hospital
RDT: Rapid diagnostic test
BF: Thick blood film
WHO: World Health Organisation
LLN: Long-lasting insecticidal mosquito nets
IRS: Indoor residual spraying
COVID 19: Coronavirus 19
SOE: State of emergency

## References

[1] Wu L, Mwesigwa J, Affara M, Bah M, Correa S, Hall T (2020). Sero-epidemiological evaluation of malaria transmission in The Gambia before and after mass drug administration. BMC Med.

[2] WHO. Guidelines for malaria – 31 March 2022. 2022. Geneva: World Health Organization.

[3] Snow RW, Amratia P, Kabaria CW, Noor AM (2012). The changing limits and incidence of malaria in Africa. Adv Parasitol.

[4] Id MH, Nwakanma D, Assogba BS, Ndiath O, Alessandro UD, Afrane YA (2021). Influence of insecticide resistance on the biting and resting preferences of malaria vectors in the Gambia. PLoS ONE.

[5] Republic of The Gambia. Malaria indicator survey. Banjul; September 2018.

[6] Ceesay SJ, Casals-pascual C, Nwakanma DC, Walther M, Gomez- N, Fulford AJC (2010). Continued decline of malaria in The Gambia with implications for elimination. PLoS ONE.

[7] Oesterholt M, Bousema JT, Mwerinde OK, Harris C, Lushino P, Masokoto A (2006). Spatial and temporal variation in malaria transmission in a low endemicity area in northern Tanzania. Malar J.

[8] Carter R, Mendis KN, Roberts D (2000). Spatial targeting of interventions against malaria. Bull World Health Organ.

[9] Boushab BM, Salem M, Ahmedou O, Ould A, Salem M, Parola P (2021). Clinical features and mortality associated with severe malaria in adults in Southern Mauritania. Trop Med Infect Dis.

[10] Yusuph R, Sawe HR, Nkondora PN, Mfinanga JA (2019). Profile and outcomes of patients with acute complications of malaria presenting to an urban emergency department of a tertiary hospital in Tanzania. BMC Res Notes.

[11] Of I, In LM, Children A (1995). Indicators of life-threatening malaria in african children.N Engl. J Med.

[12] Jallow M, Casals-pascual C, Ackerman H, Walther B, Walther M, Pinder M (2012). Clinical features of severe malaria associated with death: a 13-year observational study in The Gambia. PLoS ONE.

[13] González A, Nicolás JM, Muñoz J, Castro P, Mas J, Valls ME (2009). Severe imported malaria in adults: retrospective study of 20 Cases. Am J Trop Med Hyg.

[14] Marks ME, Armstrong M, Suvari MM, Batson S, Whitty CJM, Chiodini PL, et al. Severe imported falciparum malaria among adults requiring intensive care: a retrospective study at the hospital for tropical diseases, London. BMC Infect Dis. 2013;13:118. 10.1186/1471-2334-13-118 PMC3599148 23497139

[15] Msangeni HA, Kamugisha ML, Sembuche SH, Malecela EK, Akida JA, Temba FF (2011). Prospective study on severe malaria among in-patients at Bombo regional hospital, Tanga. BMC Infect Dis.

[16] Abdallah TM, Abdeen MT, Ahmed IS, Hamdan HZ, Magzoub M, Adam I (2013). Severe *Plasmodium falciparu*m and *Plasmodium vivax* malaria among adults at Kassala Hospital, eastern Sudan. Malar J.

[17] United Nations. Definition of youth. 2014. https://www.un.org/esa/socdev/documents/youth/fact-sheets/youth-definition.pdf.

[18] Lowe M (2020). Examining the perceptions and behaviors of Gambian adults in response to COVID-19 social mitigation strategies. Pan Afr Med J.

[19] Heuschen AK, Lu G, Razum O, Mumin AA, Sankoh O, Seidlein L, Von (2021). Public health relevant consequences of the COVID 19 pandemic on malaria in sub Saharan Africa: a scoping review. Malar J.

[20] Kigozi SP, Kigozi RN, Epstein A, Mpimbaza A, Sserwanga A, Yeka A (2020). Rapid shifts in the age specific burden of malaria following successful control interventions in four regions of Uganda. Malar J.

[21] Hoogen LL, Van Den, Griffin JT, Cook J, Sepúlveda N, Corran P, Conway DJ (2015). Serology describes a profile of declining malaria transmission in Farafenni, The Gambia. Malar J.

[22] Satoguina J, Walther B, Drakeley C, Nwakanma D, Oriero EC, Correa S (2009). Comparison of surveillance methods applied to a situation of low malaria prevalence at rural sites in The Gambia and Guinea Bissau. Malar J.

[23] Nkumama IN, Meara WPO, Osier FHA (2020). Changes in malaria epidemiology in Africa and new challenges for elimination measuring malaria. Trends Parasitol.

[24] Segula D, Frosch AP, Sanjoaquin M, Taulo D, Skarbinski J, Mathanga DP (2014). Prevalence and spectrum of illness among hospitalized adults with malaria in Blantyre, Malawi. Malar J.

[25] Sagaki P, Thanachartwet V, Desakorn V, Sahassananda D, Chamnanchanunt S, Chierakul W (2013). Clinical factors for severity of *Plasmodium falciparum* malaria in hospitalized adults in Thailand. PLoS ONE.

[26] Bruneel F, Tubach F, Corne P, Megarbane B, Mira J, Peytel E (2010). Severe imported falciparum malaria: a cohort study in 400 critically ill adults. PLoS ONE.

[27] Robinson T, Mosha F, Grainge M, Madeley R, Christian K, Centre M (2006). Indicators of mortality in African adults with malaria. Trans R Soc Trop Med Hyg.

[28] Kamau A, Mtanje G, Mataza C, Mwambingu G, Mturi N, Mohammed S (2020). Malaria infection, disease and mortality among children and adults on the coast of Kenya. Malar J.

[29] Ejov MN, Tun T, Aung S, Lwin S, Sein K (1999). Hospital -based study of severe malaria and associated deaths in Myanmar. Bull World Heath Organ.

